# Inhibition of Matrix Metalloproteinase-8 Protects Against Sepsis Serum Mediated Leukocyte Adhesion

**DOI:** 10.3389/fmed.2022.814890

**Published:** 2022-01-25

**Authors:** Xiao Fang, Shu-Fang Duan, Zhi-Yuan Hu, Jun-Jie Wang, Le Qiu, Fei Wang, Xu-Lin Chen

**Affiliations:** Department of Burns, The First Affiliated Hospital of Anhui Medical University, Hefei, China

**Keywords:** sepsis, MMP8, leukocyte adhesion, vascular injury, critical burns

## Abstract

**Purpose:**

Leukocyte adhesion to vascular and matrix Metalloproteinase-8 (MMP8) expression is increased in sepsis and associated with poor prognosis in sepsis patients. This study aimed to investigate the role of MMP8 in sepsis serum mediated leukocyte adhesion.

**Methods:**

Bioinformatics analysis of GSE64457 and GSE65682 was performed to evaluate the role of MMP8 in the progression of sepsis. Expression of MMP8 in blood samples from patients with sepsis was detected by qRT-PCR and ELISA. Human umbilical vein endothelial cells (HUVECs) were treated with sepsis serum, control serum, and MMP8 inhibitor. Expression of vascular cell adhesion molecule-1 (VCAM-1) and intercellular cell adhesion molecule-1 (ICAM-1) were detected by qRT-PCR and ELISA, respectively. The protein expression of total p38, phosphorylated-p38, ERK1/2, and p-ERK1/2 was detected by Western blotting. Peripheral blood mononuclear cells (PBMCs) and polymorphonuclear neutrophils (PMNs) were incubated with the treated HUVECs to calculate leukocyte adhesion.

**Results:**

Four hundred and twenty-nine differentially expressed genes (DEGs) and seven hub genes between sepsis patients and healthy controls were identified. GO function analysis of DEGs and hub genes indicated that the DEGs and hub genes were mainly enriched in neutrophil activation. MMP8 was selected as a key gene with an unfavorable prognosis in sepsis patients. The mRNA and protein expression of MMP8 in blood from sepsis patients were significantly higher than controls. Leukocyte adhesion and mRNA and protein expression of VCAM-1 and ICAM-1 were significantly increased in the sepsis serum group compared to that in the control group, as was the protein expression of p-p38 and p-ERK1/2. However, the MMP8 inhibitor suppressed the leukocyte adhesion promoted by sepsis serum by decreasing the expression of VCAM-1, ICAM-1, p-p38, and p-ERK1/2.

**Conclusion:**

Our study indicated that MMP8 acts as a key gene in the development of sepsis, and sepsis serum promotes leukocyte adhesion to HUVECs via MMP8, which suggest that MMP8 might be a potential therapeutic target for sepsis.

## Introduction

Sepsis, redefined in the Third International Consensus in 2016 as a life-threatening organ dysfunction caused by a dysregulated host response to infection, is a complex clinical syndrome with extensive biochemical and physiological abnormalities (1). Globally, sepsis is a major public health concern with a high incidence and mortality, affecting ~49 million people and resulting in 11 million deaths every year (2). The Dysregulated innate and adaptive immune responses to infection play crucial roles in this syndrome (3). Leukocytes are essential components of host immune cells in response to infection, and the recruitment, rolling, and adhesion of leukocytes to the endothelium are anomalously increased in sepsis (4, 5). The endothelium is recognized as a key regulator of the early inflammatory response, including the recruitment, adhesion, and migration of leukocytes, and is also considered a primary organ in the occurrence and development of sepsis (6, 7). Therefore, leukocyte and endothelial dysfunction play a vital role in the pathophysiology of sepsis (8).

Matrix Metalloproteinase-8 (MMP8), also known as neutrophil collagenase or collagenase-2, is a neutrophil-derived collagenase that can cleave various collagen, and many non-collagenous substrates containing fibronectin and some cytokines (9, 10). Interestingly, *MMP8* gene expression is overexpressed in children with septic shock, and the serum MMP8 levels have been reported to be significantly higher in patients with severe sepsis than in healthy controls (11, 12). It is clear that MMP8 works as a novel inflammatory modulator in sepsis (13). In addition, the occurrence of atherosclerosis was positively associated with serum MMP8 concentration that increased the expression of vascular cell adhesion molecule-1 (VCAM-1) (14, 15). VCAM-1, expressed on the vasculature only after cytokines stimulate endothelial cells, is involved in the adhesion and transmigration of leukocytes to vascular endothelial cells (16–18).

Considering the ability of MMP8 in upregulating the expression of VCAM-1, and the over adhesion of leukocyte to endothelium in sepsis, we hypothesized that sepsis serum promotes leukocyte adhesion to endothelium via MMP8 in sepsis. In the present study, we found that *MMP8* acts as a key gene in the progression of sepsis and is associated with an unfavorable prognosis in patients with sepsis. More importantly, in an *in vitro* study, the serum from sepsis patients can upregulate the expression of VCAM-1 and intercellular cell adhesion molecule-1 (ICAM-1) in human umbilical vein endothelial cells (HUVECs) and improve the adhesion of leukocytes to HUVECs through the phosphorylation of p38 and ERK1/2 in signal transduction. The MMP8 specific inhibitor can suppress this process.

## Materials and Methods

### Reagents

Human Neutrophil Isolation kit (#P9040) was purchased from Solarbio (Beijing, China). HUVEC (#BNCC249736) was purchased from BN-Bio (Beijing, China). MMP8 inhibitor (M8I, #sc-311436) was purchased from Santa Cruz (California, USA). Prime-ScriptTMRT regent kit (#RR047A) was purchased from TaKaRa (Shiga, Japan). Novostart SYBR qPCR SuperMix Plus (#E096-01B) was purchased from Novoprotein (Shanghai, China). Human MMP8 (#ab219050) ELISA kit was purchased from Abcam (Shanghai, China). Human VCAM-1 (#EK190-03) ELISA kit was purchased from MultiSciences (Hangzhou, China). Human ICAM-1 (#RX105101H) ELISA kit was purchased from Ruixin Biotech (Quanzhou, China). RIPA (#P0013B), PMSF (#ST505), phosphatase inhibitor (#P1050), BCA Protein Assay kit (#P0012), and ECL detection kit (#P0018FS) were purchased from Beyotime (Shanghai, China) Mouse anti-β-actin monoclonal primary antibody (#TA-09), HRP-conjugated goat anti-mouse (#ZB-2305) and goat anti-rabbit (#ZB-2301) secondary antibodies were purchased from ZSGB-Biotech (Beijing, China). Rabbit anti-p38 polyclonal (#AF6456), rabbit anti-phosphorylated p38 (p-p38) polyclonal (#AF4001), mouse anti-ERK1/2 monoclonal (#BF8004), and rabbit anti-p-ERK1/2 (#AF1015) polyclonal prinary antibody were purchased from Affinity Biosciences (Cincinnati, USA).

### Gene Expression Microarray Data

The microarray expression profiles of sepsis patients were obtained from the Gene Expression Omnibus database (http://www.ncbi.nlm.nih.gov/geo) in National Center for Biotechnology Information. The GSE64457 dataset, based on the platform of GPL570, contains 15 samples from septic shock patients and eight samples from healthy controls, which were deeply analyzed to acquire the hub genes involved in the development of sepsis (19). The GSE65682 dataset, based on the GPL13667 platform, contains samples from sepsis patients and healthy controls (20). The sepsis samples were collected from survivors and non-survivors and used as survival data for survival analysis in this study.

### Identification of DEGs and Hub Genes

The following process was conducted using specific packages in the R software (version 3.61), as previously described (21). The data were normalized after the conversion of probe sets into gene symbols, fold change (FC) of each gene was subsequently calculated using the linear models for microarray data (LIMMA) package, and differentially expressed genes (DEGs) were selected according to the criteria of |log2FC| > 1 and adj. *p* < 0.05. The DEGs are shown in a heatmap and volcano plot by using the ggplot2 package. A protein-protein interaction (PPI) network was constructed in the Search Tool for the Retrieval of Interacting Genes database (STRING database, version 11.0, http://string-db.org) after the upload of DEGs into the database and visualized in Cytoscape (version 3.7.2). According to three algorithms [maximum neighborhood component (MNC), maximal clique centrality (MCC), and degree] in the cytoHubba plugin in Cytoscape, the top 10 genes were characterized and overlapped to identify the hub genes.

### GO Functional Analysis of DEGs and Hub Genes

The potential functions of DEGs and hub genes were predicted by GO functional analysis and visualized using the ClusterProfiler package in R.

### Survival Analysis

The GSE65682 dataset was chosen for survival analysis to analyze the relationship between the expression of hub genes and the overall survival of patients. GSE65682 is the largest dataset, which contains 479 sepsis patients, with the expression data of each gene and prognosis data of each patient. The best cut-off of the gene expression value for survival analysis was determined by the surv_cutpoint function of the survminer package in R (22). The Survival analysis of high and low of each hub gene and survival curve was conducted using the survival package in R.

### Sample Collection

The study was conducted according to the guidelines of the Declaration of Helsinki, and approved by the Ethics Committee of Anhui Medical University, and all volunteers and patients provided informed consent. This study recruited six adult patients with sepsis from the Department of Intensive Care Units in the First Affiliated Hospital of Anhui Medical University, fulfilling the Sepsis-3 consensus definitions and not meeting any one of the exclusion criteria, which included malignancies, autoimmune diseases, severe systemic diseases, acute cardiovascular and cerebrovascular diseases, and pregnant and lactating women (1). Sepsis blood samples were collected within 24 h of sepsis confirmation. Healthy control blood samples were collected from five adult healthy controls recruited from the Department of Physical Examination Center without any diseases. The sera of sepsis patients and healthy controls were separated and stored at −80°C until use. Normal peripheral blood mononuclear cells (PBMCs) and polymorphonuclear neutrophils (PMNs) were separated from the blood samples of healthy controls by gradient density centrifugation according to the manufacturer's instructions of the Human Neutrophil Isolation kit before use.

### Cell Culture and Treatments

The HUVEC complete medium (#CM-0122) containing 10% FBS and other essential factors was purchased from Procell (Wuhan, China). The serum from sepsis patients and healthy controls were pooled together separately before use and blended with a complete medium in a proportion of 1:4 to test the effect of sepsis patients' serum on leukocyte adhesion. To test the effect of M8I, 20 μM M8I, as used in previous study, was added to the complete medium for 1 h in advance and then, into the mixed medium for continuous culture (23). After HUVECs were cultured in different mixed media under 5% CO2 at 37°C for 6 h, the supernatant and cells were collected for the following test.

### Quantitative Real-Time PCR (qRT-PCR)

According to the manufacturer's instructions, the total RNA of separated PMNs and cultured cells was extracted using TRIzol and immediately transcribed into cDNA by using the Prime-ScriptTMRT reagent kit. qRT-PCR was performed using synthesized cDNA, specific primers (Table 1), and Premix EX Taq. The mRNA expression of genes was calculated using the 2^−ΔΔCt^ method, which was normalized to *GAPDH*.

**Table 1:** Sequence of primers for Real-Time RT-PCR.

**Genes**	**Forward primer**	**Reverse primer**
*MMP8*	GCTTTCAGGGAAACCAGCAA	TGTGCTTGGTCCAGTAGGTT
*VCAM-1*	AGATGGCGCCTATACCATCC	TAGAGCACGAGAAGCTCAGG
*ICAM-1*	CACAGTCACCTATGGCAACG	GTCCCTTCTGAGACCTCTGG
*GAPDH*	CGGATTTGGTCGTATTGG	GGTGGAATCATATTGGAACA

### Enzyme-Linked Immunosorbent Assay (ELISA)

According to the instructions of the ELISA kit, the level of MMP8 in the serum of patients and controls, and the levels of VCAM-1 and ICAM-1 in the culture supernatant were determined, and the optical density was measured on a spectrophotometer at 450 nm.

### PBMC and PMN Cell Adhesion

After the differentially treated cells were incubated with counted fresh PBMCs and PMNs for 2 h, the PBMCs and PMNs were washed and counted. The difference between the counts of fresh and washed cells was considered to be the number of adhesive cells. The cell adhesion rate was counted as the ratio adhesive cells in different group ratios to the adhesive cells in the control group.

### Western Blotting Assay

Cells were collected and treated with RIPA buffer mixed with PMSF and phosphatase inhibitors to separate total proteins. After the protein concentration was detected using the BCA Protein Assay kit, 20 μg of total protein was transferred from 10% SDS-PAGE to a PVDF membrane. The membranes were blocked in 5% skim milk at 20°C for 2 h and incubated at 4°C overnight with the following primary antibodies: β-actin (1:1000), p38 (1:1000), p-p38 (1:1000), ERK1/2 (1:1000), and p-ERK1/2 (1:1000). After incubation with goat anti-mouse (1:10000) and goat anti-rabbit (1:10000) secondary antibodies at 20°C for 2 h, the protein bands were visualized using an ECL detection kit, and the gray value of the bands was quantified using ImageJ (version1.8.0) and finally normalized to β-actin.

### Statistical Analysis

The *p*-value of each gene was calculated and converted to an adjusted *p*-value by false discovery rate correction of Benjamini and Hochberg test in identification of DEGs by using the LIMMA package in R. All the experimental data conformed to normal distribution and were presented as mean ± standard error of the mean, and comparisons between two groups were performed using an unpaired *t*-test. Statistical significance was set at *p* < 0.05. Statistical analysis of data and graph development was performed using GraphPad Prism (version 8.0).

## Results

### Identification of DEGs and Hub Genes

**Figure 1:**
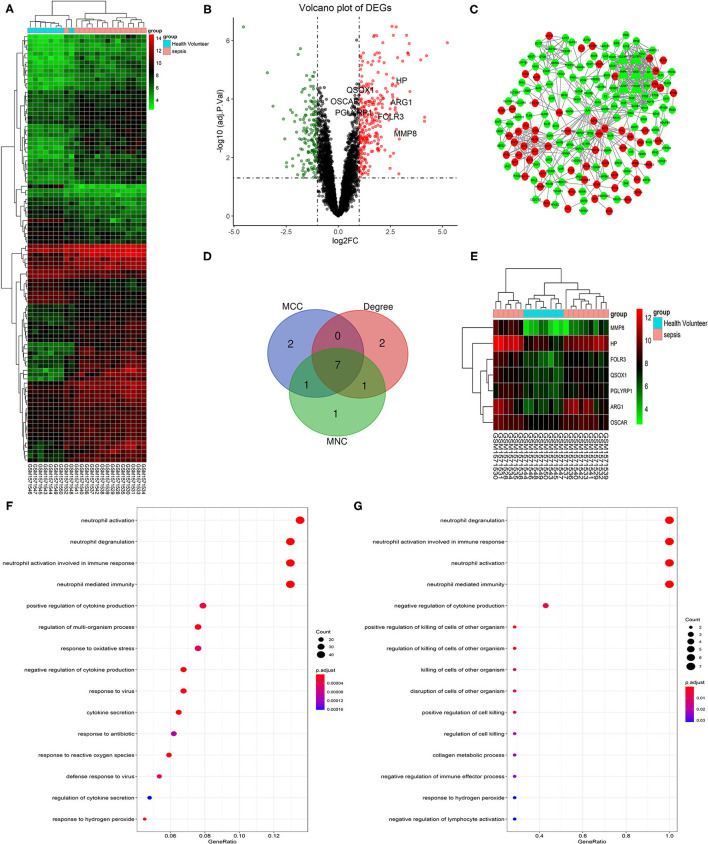
Identification and GO functional analysis of DEGs and hub genes in GSE64457. **(A)** The top 100 most significant DEGs are shown in the heatmap (|log2FC| > 1 and adj. *p* < 0.05). Red indicates a high expression, while green indicates a relatively low expression. **(B)** The volcano plot shows the 429 DEGs that containing 266 upregulated genes in red (log2FC > 1 and adj. *p* < 0.05) and 163 downregulated genes in green (log2FC < −1 and adj. *p* < 0.05). Hub genes are highlighted in the plot. **(C)** Shows the PPI network of DEGs. Red notes the upregulated genes and green noted the downregulated genes. Triangles represent the hub genes in the network. **(D)** Hub genes (*MMP8, HP, ARG1, FOLR3, QSOX1, PGLYRP1, OSCAR*) were identified by the overlapping of three algorithms (MNC, MCC, and Degree) in the cytoHubba plugin in Cytoscape and clustered in heatmap **(E)**. The top 15 GO terms are shown in the dot plots of GO functional analysis of DEGs **(F)** and hub genes **(G)** (*p* < 0.05). The size of each circle is positive correlated with the counts of the enriched genes in this term. GO, gene ontology; DEGs, differentially expressed genes; PPI, protein-protein interaction; MMP8, matrix metallopeptidase 8; HP, haptoglobin; ARG1, arginase 1; FOLR3, folate receptor gamma; QSOX1, quiescin sulfhydryl oxidase 1; PGLYRP1, peptidoglycan recognition protein 1; OSCAR, osteoclast associated Ig-like receptor; MNC, maximum neighborhood component; MCC, maximal clique centrality.

According to the screening criteria: |log2FC| > 1 and adj. *p* < 0.05, a total of 429 DEGs containing 266 upregulated genes and 163 downregulated genes were identified between sepsis patients and healthy controls. The top 100 DEGs were clustered in a heatmap (Figure 1A), and the up-and down-regulated genes were separated in a volcano plot (Figure 1B). The PPI network was constructed using the total uploaded DEGs in the STRING database and visualized in Cytoscape (Figure 1C). The top 10 key genes were selected by three algorithms (MNC, MCC, and degree) in the cytoHubba plugin in Cytoscape and overlapped to identify the seven hub genes (*MMP8, HP, ARG1, FOLR3, QSOX1, PGLYRP1*, and *OSCAR*; Figures 1D,E). Fold changes in the seven hub genes between sepsis and control in GSE64457 are shown in Table 2.

**Table 2:** LogFC and adj. *p* Val of 7 hub genes in dataset GSE64457.

**Gene**	**logFC**	**adj. *p*. Val**
*MMP8*	2.929935453	0.002329725
*HP*	2.753424366	3.57E-05
*ARG1*	2.739062931	0.000198584
*FOLR3*	1.779352541	0.000226394
*QSOX1*	1.362309413	3.06E-05
*PGLYRP1*	1.328988662	0.000153406
*OSCAR*	1.141676879	0.000136499

### GO Functional Analysis of DEGs and Hub Genes

GO functional analysis of DEGs and hub genes was performed using ClusterProfiler in R to understand their potential functions. The top four enriched terms were neutrophil activation, neutrophil activation involved in the immune response, neutrophil degranulation, and neutrophil-mediated immunity (Figure 1F). Similarly, the top four terms of hub genes enriched were neutrophil degranulation, neutrophil activation, neutrophil activation involved in immune response, and neutrophil-mediated immunity (Figure 1G).

### Survival Analysis of Hub Genes

**Figure 2:**
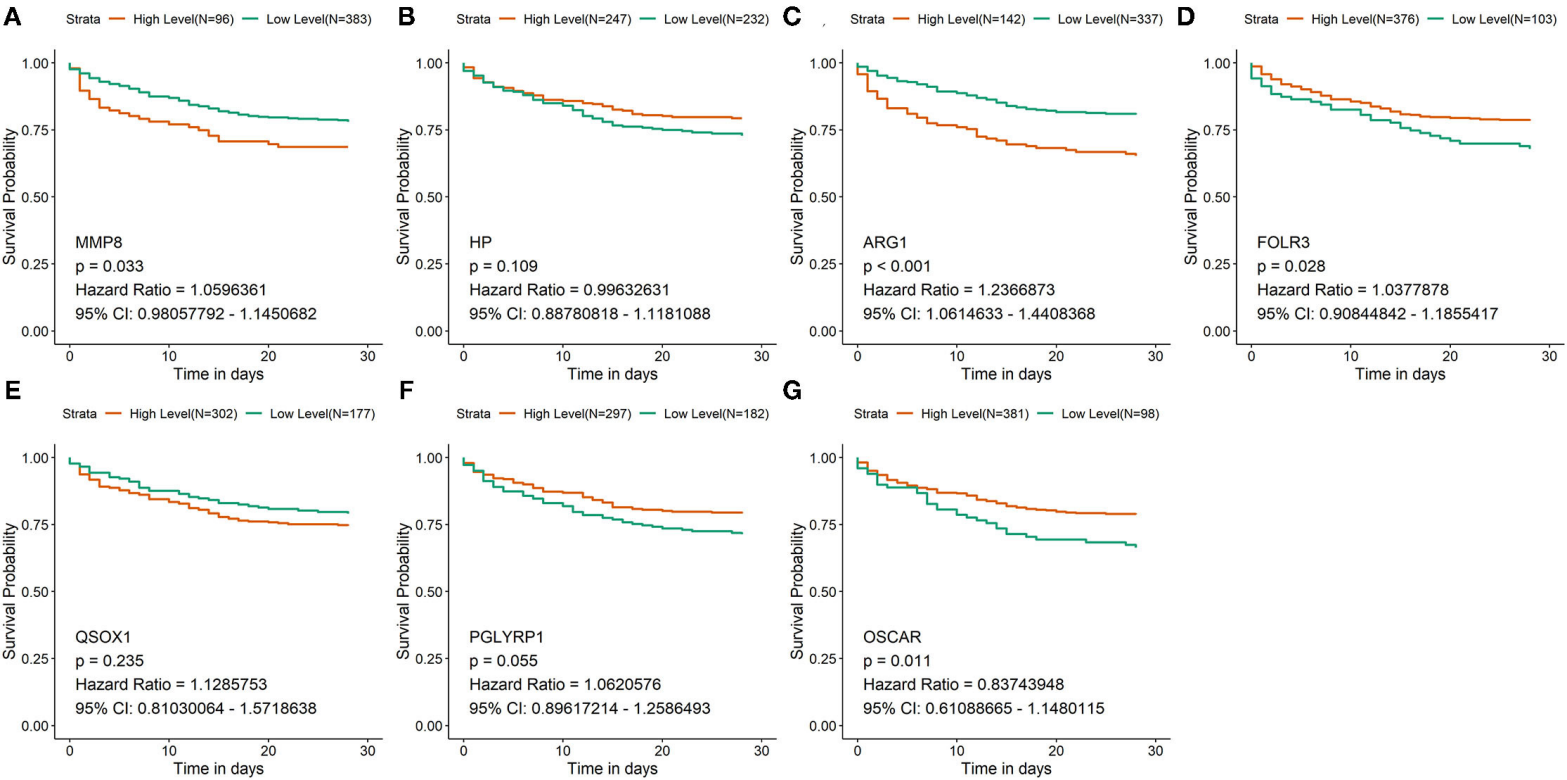
Survival analysis of hub genes in GSE65682. **(A–G)** The survival curves of the 7 hub genes in GSE65682. Patients with high expression of *FOLR3* and *OSCAR* had a better prognosis than low expression of these genes. Patients with low expression of *MMP8* and *ARG1* had a better prognosis than high expression of these genes.

According to the expression of each hub gene, sepsis patients in GSE65682 were separately divided into high-expression and low-expression groups by using the surv_cutpoint function of the Survminer package. Survival analysis was subsequently performed in R to analyze the relationship between the expression of hub genes and the overall survival of patients (Figures 2A–G). According to the survival analysis, patients with high expression of *FOLR3* (hazard ratio [HR] = 1.038, *p* < 0.05) and *OSCAR* (HR = 0.837, *p* < 0.05) had a better prognosis than those with low expression of these genes, and patients with high expression of *MMP8* (HR = 1.060, *p* < 0.05) and *ARG1* (HR = 1.237, *p* < 0.05) had an unfavorable prognosis compared to those with low expression. Expression of HP, QSOX1 (HR = 1.129, *p* > 0.05), and *PGLYRP1* (HR = 1.062, *p* > 0.05) did not have a statistically significant predictive effect on the survival of patients with sepsis.

### Expression of MMP8 in Sepsis Patients

**Figure 3:**
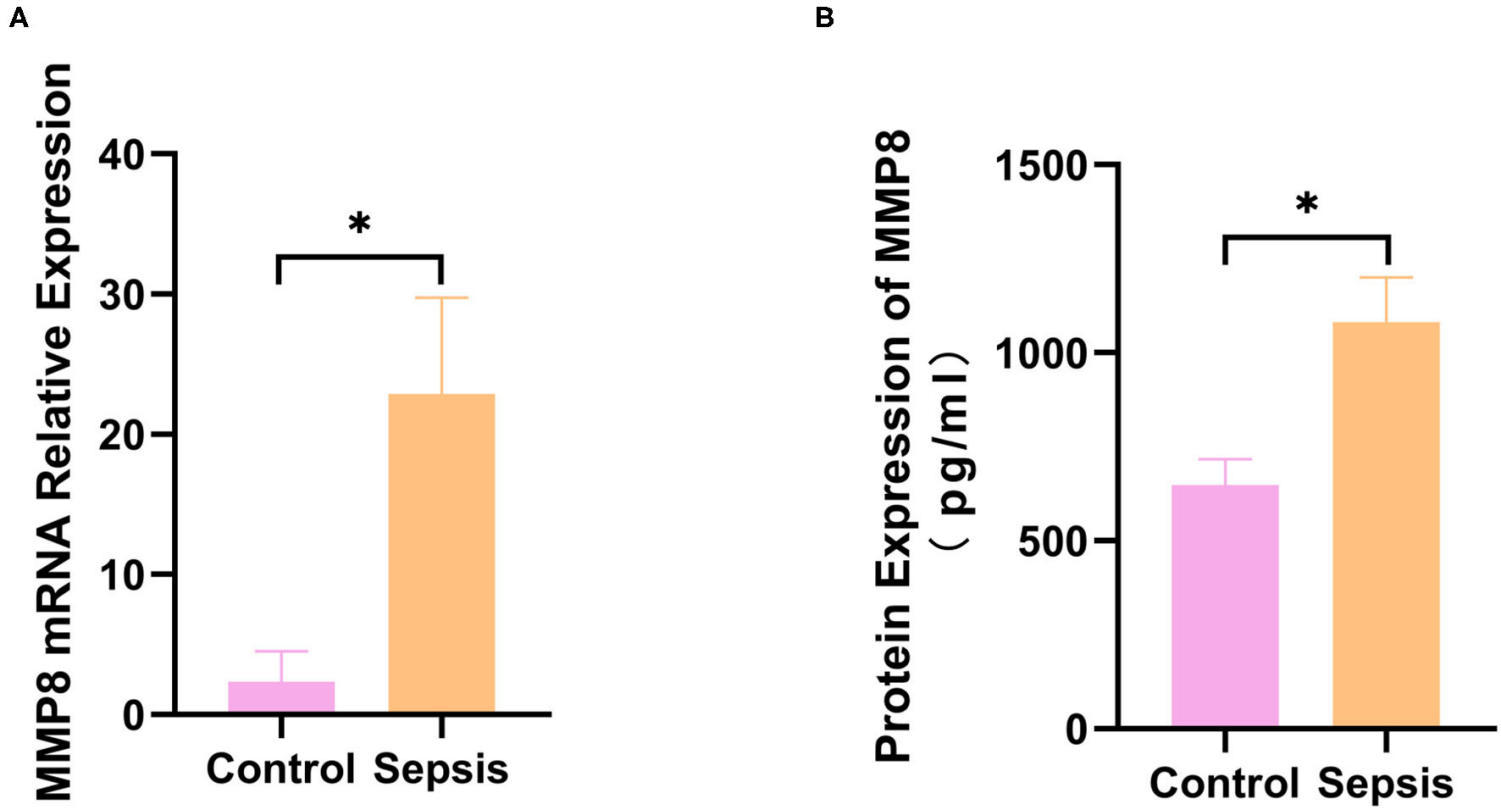
Expression of MMP8 in blood samples from sepsis patients and controls. **(A)** mRNA expression of MMP8 in neutrophil of patients and controls was measured by q-RTPCR. **(B)** Protein level of MMP8 in serum of patients and controls was detected by ELISA. *p < 0.05 between control and sepsis group by using *t*-test.

Thus, *MMP8* is considered a key gene in sepsis development. The gene and protein expression of MMP8 were validated by qRT-PCR and ELISA in six sepsis patients and five healthy controls. Gene expression (2.360 ± 0.966 vs. 22.900 ± 6.845, *p* < 0.05, Figure 3A) and protein expression (647.6 ± 69.37 vs. 1081 ± 119.3, *p* < 0.05, Figure 3B) of MMP8 was significantly higher in sepsis patients than in healthy controls.

### Sepsis Serum Promotes Leukocyte Adhesion to HUVEC

**Figure 4:**
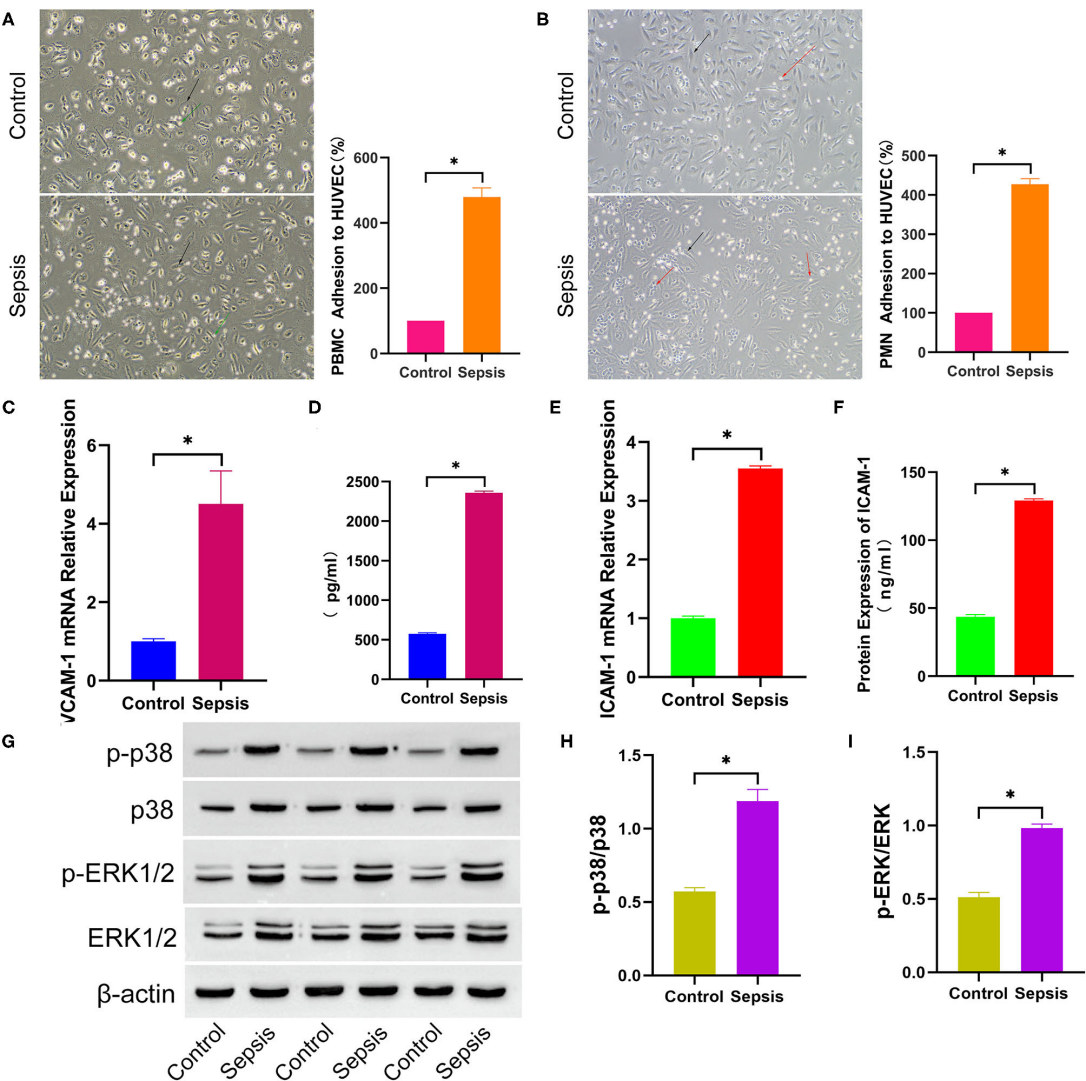
Sepsis serum promotes leukocyte adhesion to HUVEC. After incubated with differently treated HUVEC for 2 h, PBMC **(A)** and PMN **(B)** adhesion to HUVEC were shown in the bright field picture and measured by calculating the difference of cell counts between incubated leukocyte and washed leukocyte. The black arrow indicates HUVEC, green arrow indicates PBMC, and red arrow indicates PMN. After HUVEC treated with sepsis serum and control serum for 6 h separately, the mRNA expression of VCAM-1 **(C)** and ICAM-1 **(E)** were measured by q-RTPCR, and the protein level of VCAM-1 **(D)** and ICAM-1 **(F)** in supernatant were detected by ELISA. **(G)** The protein expression of total p38, p-p38, total ERK1/2, and p-ERK1/2 between sepsis group and control group was tested by western blotting assay. The relative gray value is shown in histogram **(H,I)**. *p < 0.05 between control and sepsis group by using *t*-test.

To explore the effect of sepsis serum on leukocyte adhesion to HUVECs, HUVECs were cultured in medium mixed with sepsis serum or control serum for 6 h. After the differentially treated cells were continuously incubated with counted fresh PBMCs and PMNs for 2 h, the PBMCs and PMNs were washed and counted. PBMC adhesion (100 vs. 479.3 ± 27.69, *p* < 0.05, Figure 4A) and PMN adhesion (100 vs. 427.3 ± 14.15, *p* < 0.05, Figure 4B) in the sepsis serum group was significantly higher than that in the control group. As the adhesion of leukocytes to endothelial cells is commonly mediated by VCAM-1 and ICAM-1, the supernatant and cells were collected to detect VCAM-1 and ICAM-1 further after HUVECs were cultured in a mixed medium for 6 h. VCAM-1 (1.004 ± 0.06 vs. 4.508 ± 0.840, *p* < 0.05, Figure 4C) and ICAM-1 (1.001 ± 0.04 vs. 3.552 ± 0.04, *p* < 0.05, Figure 4E) mRNA expression was significantly increased in sepsis serum-treated HUVECs compared to the control group. In addition, the protein levels of VCAM-1 (576.3 ± 11.31 vs. 2360 ± 19.72, *p* < 0.05, Figure 4D) and ICAM-1 (43.66 ± 1.69 vs. 129.2 ± 1.30, *p* < 0.05, Figure 4F) were also significantly higher in the supernatant of sepsis serum group than in controls. Moreover, Western blot analysis (Figure 4G) indicated that the protein levels of p-p38 (p-p38/p38, 0.57 ± 0.03 vs 1.19 ± 0.08, *p* < 0.05, Figure 4H) and p-ERK1/2 (p-ERK/ERK, 0.51 ± 0.03 vs 0.98 ± 0.03, *p* < 0.05, Figure 4I) were significantly higher in sepsis serum-treated HUVECs than in the control group. The unprocessed original full blot images were attached in Supplementary Materials.

### M8I Inhibits the Effect of Sepsis Serum on Promoting Leukocytes Adhesion to HUVEC

**Figure 5:**
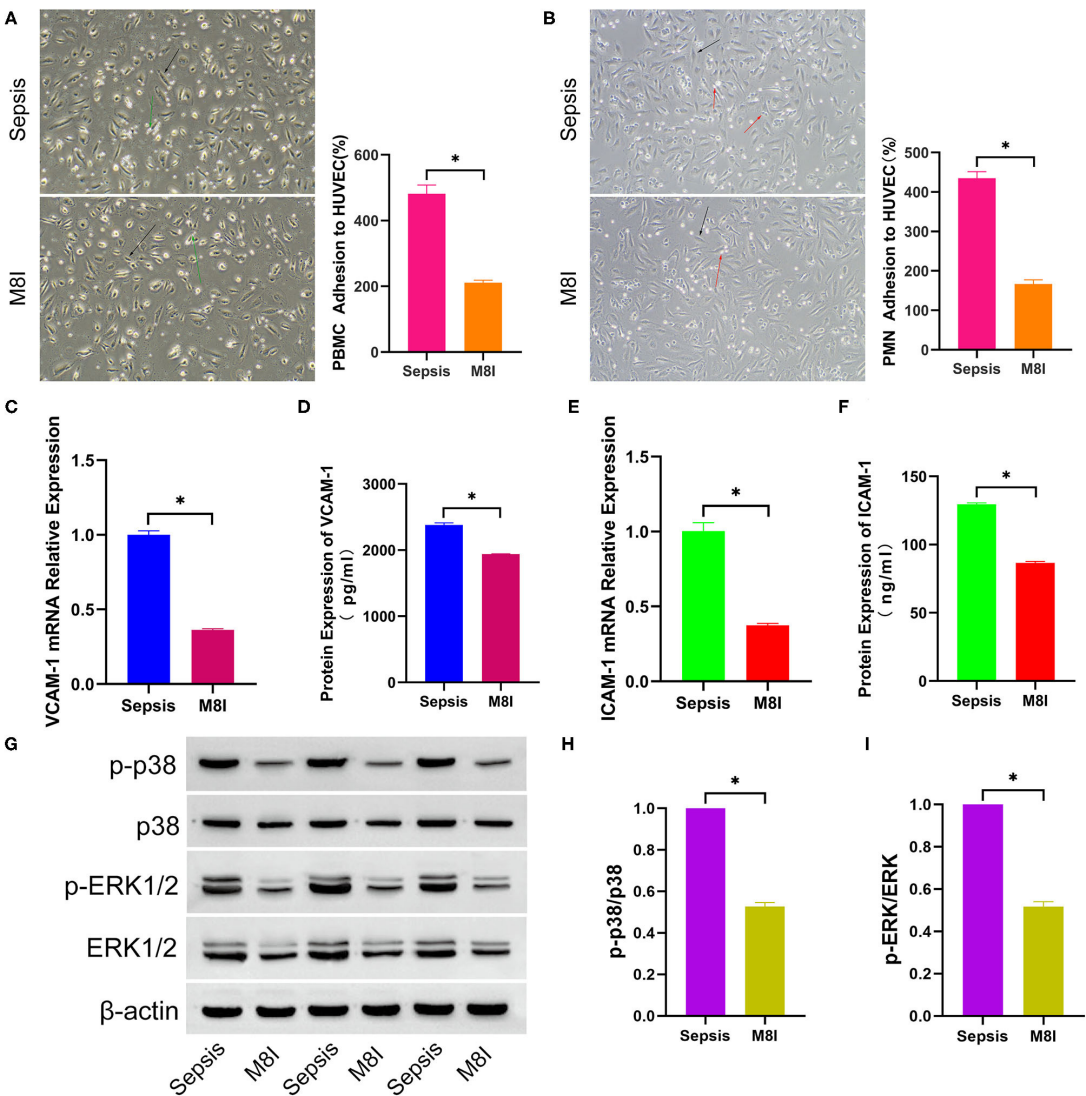
M8I Inhibits the effect of sepsis serum on promoting leukocytes adhesion to HUVEC. After incubated with differently treated HUVEC for 2 h, PBMC **(A)** and PMN **(B)** adhesion to HUVEC were shown in the bright field picture and measured by calculating the difference of cell counts between incubated leukocyte and washed leukocyte. The black arrow indicates HUVEC, green arrow indicates PBMC, and red arrow indicates PMN. After HUVEC treated with sepsis serum and M8I (20 μM) for 6 h separately, the mRNA expression of VCAM-1 **(C)** and ICAM-1 **(E)** were measured by q-RTPCR, and the protein level of VCAM-1 **(D)** and ICAM-1 **(F)** in supernatant were detected by ELISA. **(G)** The protein expression of total p38, p-p38, total ERK1/2, and p-ERK1/2 between sepsis group and control group was tested by western blotting assay. The relative gray value is shown in histogram **(H,I)**. *p < 0.05 between control and sepsis group by using *t*-test.

MMP8 plays a crucial role in the prognosis of sepsis patients based on the results of an *in silico* study, and MMP8 can promote the expression of VCAM-1 in patients with atherosclerosis. Therefore, we hypothesized that MMP8 plays a vital role in leukocyte adhesion to endothelial cells in patients with sepsis. To validate this hypothesis, M8I, a specific MMP8 inhibitor, was added to the complete medium in advance and a mixed medium for continuous culture in the M8I group. As shown in Figure 5, PBMC (481.7 ± 26.42 vs. 210.7 ± 7.88, *p* < 0.05, Figure 5A) and PMN (434.7 ± 16.90 vs. 166.7 ± 10.68, *p* < 0.05, Figure 5B) adhesion were significantly lower in M8I treated group than sepsis serum group. Compared with the sepsis serum group, the mRNA levels of VCAM-1 (1.00 ± 0.03 vs. 0.36 ± 0.01, *p* < 0.05, Figure 5C) and ICAM-1 (1.00 ± 0.06 vs. 0.37 ± 0.01, *p* < 0.05, Figure 5E) were significantly reduced after treatment with M8I. Similarly, the protein levels of VCAM-1 (2381 ± 29.31 vs. 1941 ± 3.59, *p* < 0.05, Figure 5D) and ICAM-1 (129.5 ± 1.05 vs. 86.54 ± 1.10, *p* < 0.05, Figure 5F) in the supernatant were also significantly reduced in the presence of M8I. Interestingly, the protein level (Figure 5G) of p-p38 (p-p38/p38, 1.38 ± 0.07 vs. 0.53 ± 0.02, *p* < 0.05, Figure 5H) and p-ERK1/2 (p-ERK/ERK, 1.00 ± 0.08 vs. 0.52 ± 0.02, *p* < 0.05, Figure 5I), which were examined by Western blotting, were significantly decreased after treatment with M8I.

## Discussion

Sepsis is characterized by a dysregulated host response to infection, which includes dysregulated innate and adaptive immunity (3). According to the pathophysiology of sepsis, the hyperfunction of host immunity in the early stage of sepsis and immunosuppression in the middle and end stages of sepsis causes systemic immune dysfunction, ultimately leading to organ failure (24). Peripheral blood leukocytes, mostly consisting of PMN which are the major effector cell of innate immunity, and PBMCs, the major effector cells of adaptive immunity, play crucial roles in host immunity in sepsis. Leukocyte adhesion to the vasculature and expression of VCAM-1 and ICAM-1 are associated with the development of multiple organ failure in severe sepsis (25, 26). However, whether sepsis serum promotes leukocyte adhesion remains unknown. In the present study, for the first time, HUVECs were cultured in a medium containing sepsis serum for 6 h to detect leukocyte adhesion. Adhered PBMCs and PMNs were significantly increased in sepsis serum mixed medium compared to the control, similar to the mRNA and protein levels of VCAM-1 and ICAM-1. In addition, the phosphorylation of p-p38, and p-ERK1/2 protein was also significantly increased in the sepsis serum group.

Neutrophils are the primary innate immune cells that play vital roles in host defense against infection, affect the function of T lymphocytes and B lymphocytes, and finally lead to immunosuppression in the middle and end stages of sepsis (27–29). Commonly, activated neutrophils exert their biological functions by releasing granules containing proteolytic enzymes and inflammatory mediators (30). Demarset et al. globally evaluated the alterations of phenotypic, functional, and transcriptomic studies in neutrophil of immunosuppressed septic patients (19). To further analyze the gene expression of neutrophils in sepsis-associated immunosuppression, dataset GSE64457 from Demaret J's study in the GEO database was downloaded and deeply analyzed to elucidate the GO function of DEGs and identify the hub genes. GO function analysis of DEGs and hub genes revealed that the genes were mainly enriched in neutrophil activation, neutrophil activation involved in immune response, neutrophil degranulation, and neutrophil-mediated immunity, which were consistent with the pathophysiology of sepsis (31, 32). However, the GSE64457 dataset does not have patient survival data. Therefore, GSE65682 was chosen to conduct the survival analysis of seven hub genes because it is the largest dataset that contains 479 sepsis patients with the expression data of each gene and prognosis data of each patient. In survival analysis of seven hub genes, patients with high expression of *FOLR3* and *OSCAR* had a better prognosis than those with low expression of these genes, and patients with high expression of *MMP8* and *ARG1* had an unfavorable prognosis than those with low expression of these genes. Expression of *HP, QSOX1*, and *PGLYRP1* did not have a statistically significant predictive effect on the survival of patients with sepsis.

ARG1 mainly exists in cytoplasmic azurophilic granules of neutrophils and catalyzes the hydrolysis of arginine to ornithine and urea (33). Besides, ARG1 is responsible for immune suppression by suppressing T cell functions via l-arginine depletion (34, 35). Therefore, MMP8 attracted our attention in this study. MMP8 is a neutrophil-derived collagenase that was originally thought to function primarily in the degradation of the extracellular matrix. However, an increasing number of studies have indicated that MMP8 acts as an inflammatory mediator in various inflammatory disorders (36–38). Interestingly, gene expression and protein expression of MMP8 were significantly higher in patients with severe sepsis than in healthy controls (11, 12). Moreover, the occurrence of atherosclerosis was positively associated with serum MMP8 concentration, which increased the expression of VCAM-1 (14, 15). Therefore, we hypothesized that MMP8 is an important inflammatory mediator that mediates sepsis serum-promoted leukocyte adhesion to HUVECs.

To verify this hypothesis, M8I, a specific MMP8 inhibitor, was added to the complete medium 1 h in advance and then, into the mixed medium for continuous culture. PBMC and PMN adhesion were significantly decreased in the M8I group compared to the sepsis serum group. In addition, the mRNA and protein levels of VCAM-1 and ICAM-1 were also decreased in the M8I group. Similarly, the phosphorylation of p-p38 and p-ERK1/2 protein was significantly decreased in HUVECs treated with M8I.

Leukocytes, which contain neutrophils, monocytes/macrophages, and lymphocytes, are the main effector cells in the immune response. The wide adhesion of leukocytes to the endothelium in sepsis causes a decrease in the number of circulating leukocytes; thus, the number of recruited leukocytes to the infection site also decreases, and host immunity is affected (39). The deleterious accumulation of neutrophils in the endothelial vasculature of organs leads to collateral tissue damage and, ultimately, multiple-organ failure (40). Leukopenia, which is probably caused by the over-adhesion of leukocytes to the endothelium, is detrimental to the prognosis of sepsis (41). In addition, endothelial function is also impaired by the released granules after adhesion and infiltration of leukocytes on the endothelium, which accelerates organ dysfunction (42, 43). Therefore, excessive leukocyte adhesion to endothelial vascular stimulation by MMP8 is detrimental to the survival of sepsis patients.

Although we believe that our study makes a significant contribution to the literature, this study had two limitations. First, for the spread of COVID-19 epidemic, this study lacked a rescue experiment *in vitro*. Although we used M8I to validate that MMP8 stimulates leukocyte adhesion, it is more rigorous to add recombinant human MMP8 into the M8I group. In the next step of our study, after the control of COVID-19 epidemic, we plan to investigate the exact role and the mechanism of MMP8 in leukocyte adhesion. Furthermore, the phosphorylation of p38 and ERK1/2 was empirically detected by Western blotting. p38 and ERK1/2 are the main components of the MAPK pathway, which has been identified as the major signaling pathway in inflammation and is closely associated with inflammatory regulation and control mechanisms (44). The phosphorylation of p38 and ERK1/2 commonly activates NF-κB or AP-1 transcription factors and ultimately enhances the transcription of specific inflammation-related genes (45, 46). Indeed, the phosphorylation of p38 and ERK1/2 varied with the stimulation of MMP8 in this study. We will explore the pathway by which the signal transduces from MMP8 to VCAM-1 and ICAM-1 via the phosphorylation p38 and ERK1/2 in our next study.

## Conclusion

**Figure 6:**
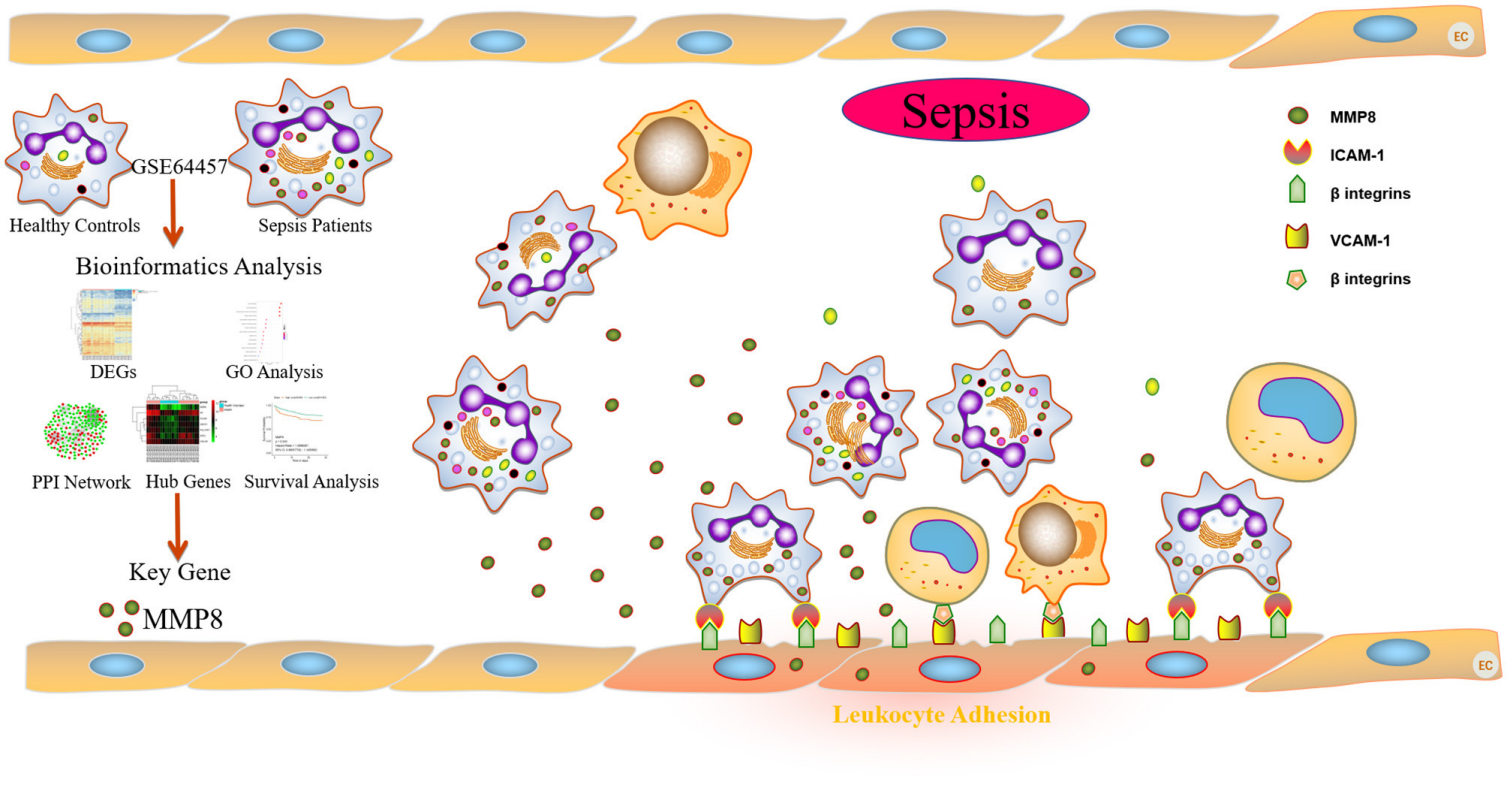
Schematic summary of the *in silico* and *in vitro* study of sepsis serum promotes leukocyte adhesion to HUVEC via MMP8.

In conclusion, as shown in Figure 6, the present study provides the first evidence that MMP8 acts as a key gene in the progression of sepsis in an *in silico* study, and the promoted effects of sepsis serum on adhesion of leukocytes to HUVECs via MMP8 in an *in vitro* study. Our results indicated that sepsis serum promoted the adhesion of leukocytes to HUVECs by improving the expression of VCAM-1 and ICAM-1. M8I suppressed this effect by inhibiting the phosphorylation of p38 and ERK1/2. Collectively, our study demonstrates the potential role of MMP8 in mediating leukocyte adhesion to endothelial cells. This study sheds light on the role of MMP8 in the development of sepsis and provides a potential therapeutic target for sepsis.

## Data Availability Statement

The datasets presented in this study can be found in online repositories. The names of the repository/repositories and accession number(s) can be found in the article/Supplementary Material.

## Ethics Statement

The studies involving human participants were reviewed and approved by the Ethics Committee of Anhui Medical University. The patients/participants provided their written informed consent to participate in this study.

## Author Contributions

X-LC developed the idea, designed the study, and provided financial support for the study. XF performed the experiment, drafted the manuscript, summarized the data, and contributed to data interpretation. S-FD, Z-YH, J-JW, LQ, and FW were involved in the acquisition of the data. X-LC had full access to all the data in the study and was responsible for submission for publication. All authors contributed to the article and approved the submitted.

## Funding

This current study was supported by grants from the National Science Foundation of China (Nos. 81671877 and 82172204).

## Conflict of Interest

The authors declare that the research was conducted in the absence of any commercial or financial relationships that could be construed as a potential conflict of interest.

## Publisher's Note

All claims expressed in this article are solely those of the authors and do not necessarily represent those of their affiliated organizations, or those of the publisher, the editors and the reviewers. Any product that may be evaluated in this article, or claim that may be made by its manufacturer, is not guaranteed or endorsed by the publisher.
