# Supplementary figures and images for: Inhibition of Matrix Metalloproteinase-8 Protects Against Sepsis Serum Mediated Leukocyte Adhesion
Source: Front Med (Lausanne). 2022 Jan 25;9:814890. doi: 10.3389/fmed.2022.814890 (PMC8821815; doi:10.3389/fmed.2022.814890)

## Slide 1
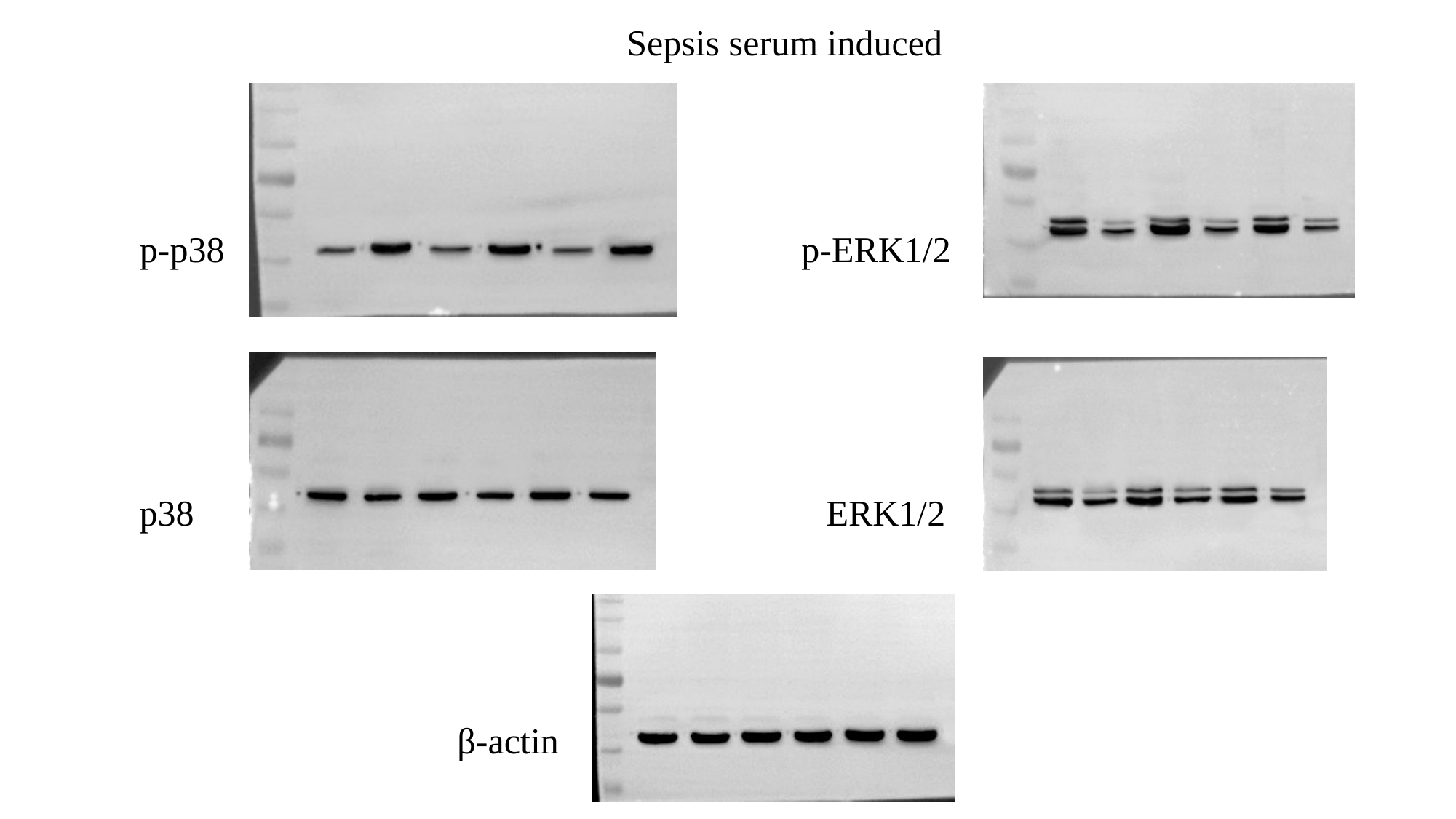

Sepsis serum induced
p-ERK1/2
p-p38
ERK1/2
p38
β-actin

## Slide 2
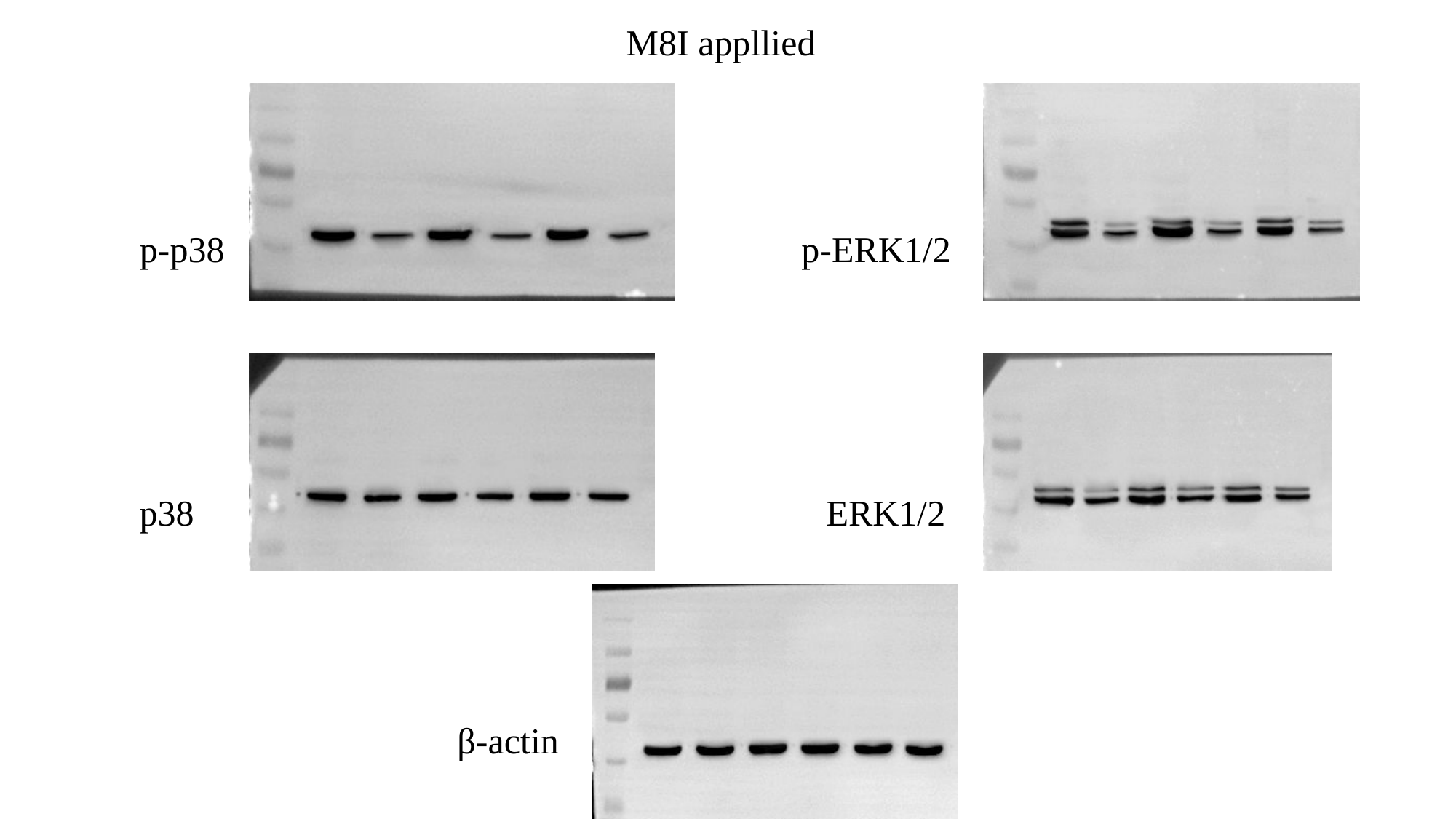

M8I appllied
p-ERK1/2
p-p38
ERK1/2
p38
β-actin

Supplement: Supplementary file 1 [file Presentation_1.PPTX]
