# Supplementary material for: Inhibition of Matrix Metalloproteinase-8 Protects Against Sepsis Serum Mediated Leukocyte Adhesion
Source: Front Med (Lausanne). 2022 Jan 25;9:814890. doi: 10.3389/fmed.2022.814890 (PMC8821815; doi:10.3389/fmed.2022.814890)
Supplement: Supplementary file 2 [file Presentation_2.PPTX]

## Slide 1
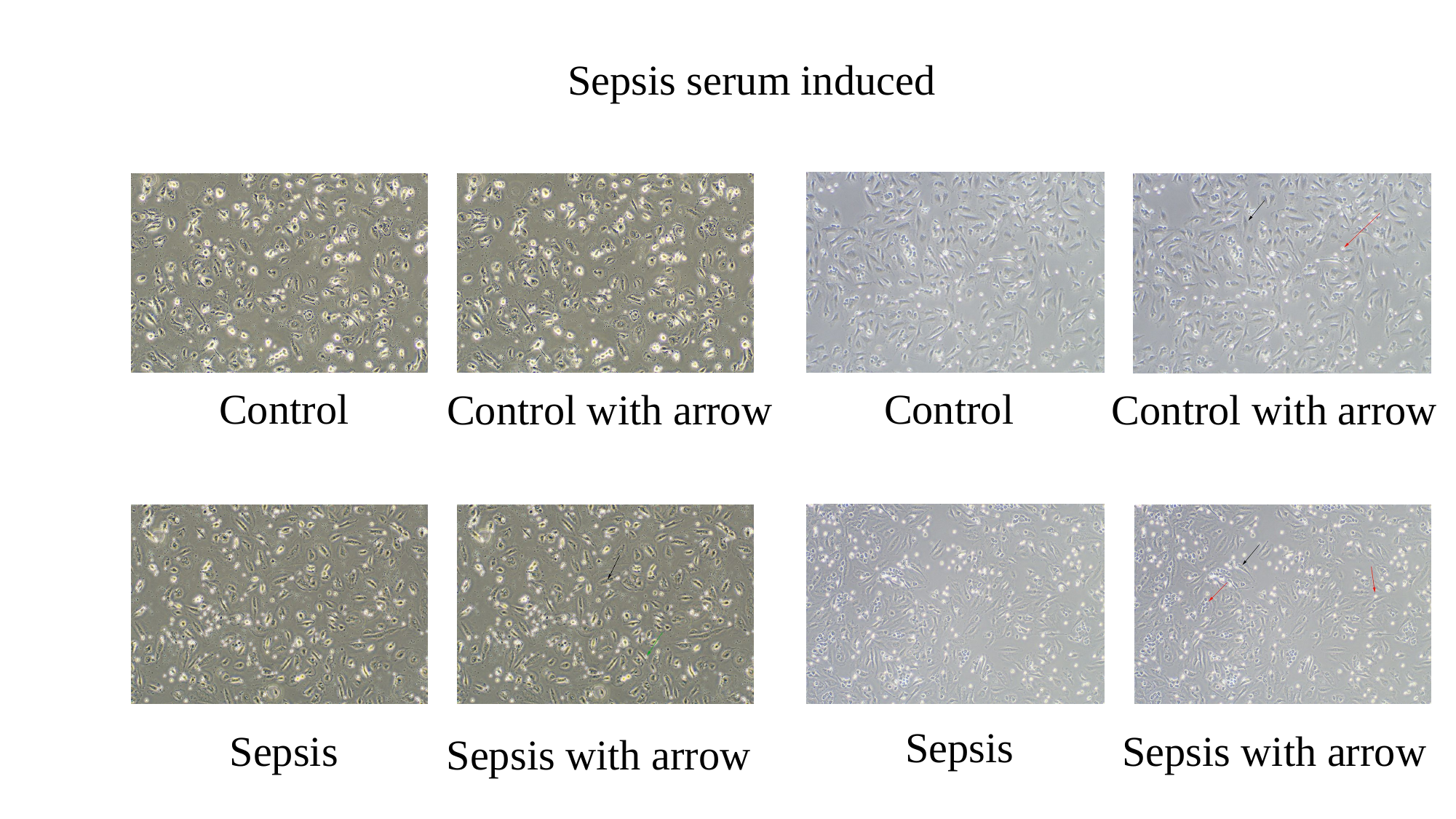

Sepsis serum induced
Control
Control
Control with arrow
Control with arrow
Sepsis
Sepsis
Sepsis with arrow
Sepsis with arrow

## Slide 2
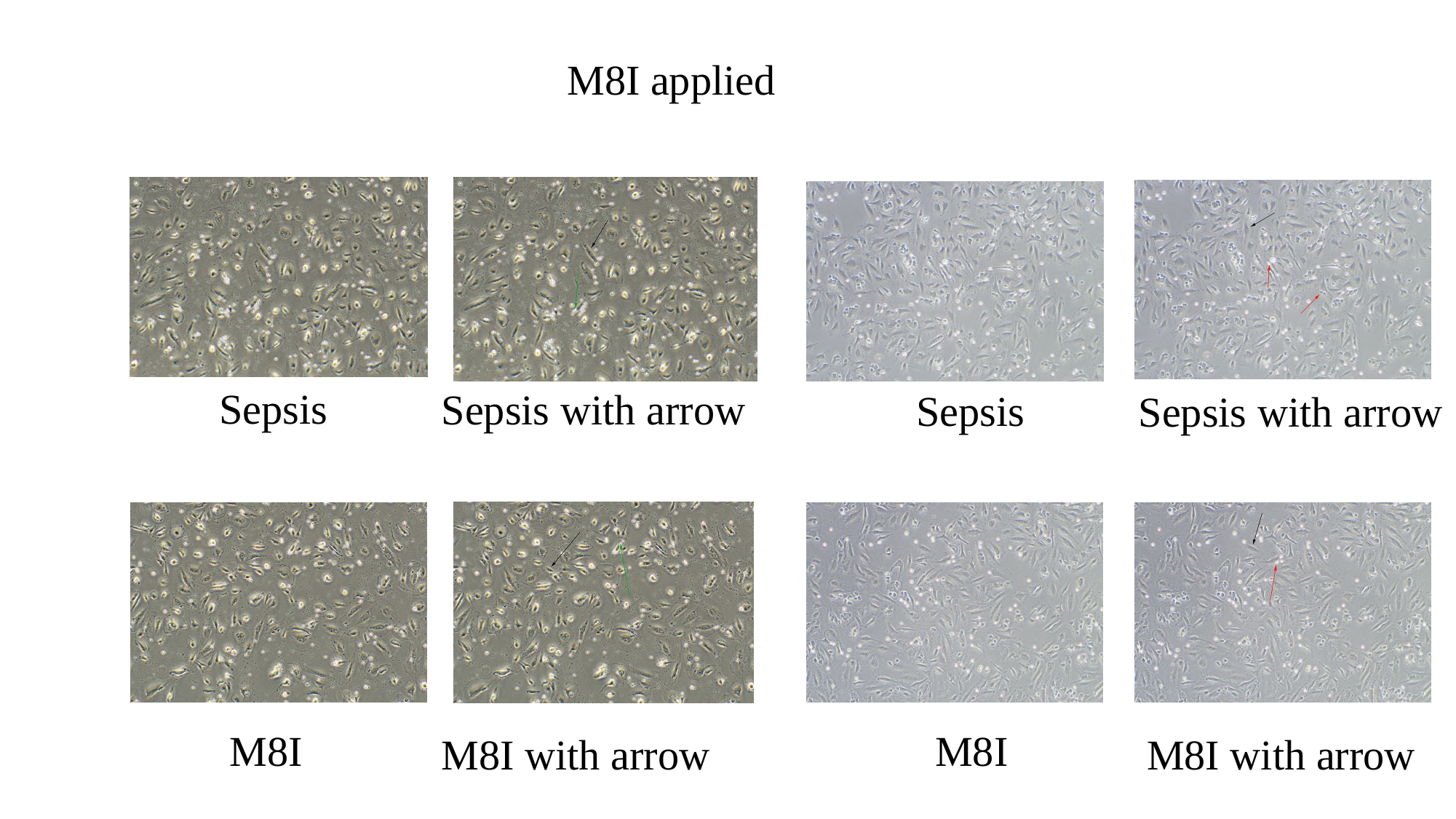

M8I applied
Sepsis
Sepsis with arrow
Sepsis
Sepsis with arrow
M8I
M8I
M8I with arrow
M8I with arrow
